# Clinical features and histological description of tongue lesions in a large Northern Italian population

**DOI:** 10.4317/medoral.20556

**Published:** 2015-08-04

**Authors:** Alessio Gambino, Mario Carbone, Paolo-Giacomo Arduino, Marco Carrozzo, Davide Conrotto, Carlotta Tanteri, Lucio Carbone, Alessandra Elia, Zaira Maragon, Roberto Broccoletti

**Affiliations:** 1 Department of Surgical Sciences, Oral Medicine Section, CIR - Dental School, University of Turin, Turin, Italy; 2 Oral Medicine Department, Centre for Oral Health Research, Newcastle University, Newcastle upon Tyne, UK; 3 Private practice, Turin

## Abstract

**Background:**

Only few studies on tongue lesions considered sizable populations, and contemporary literature does not provide a valid report regarding the epidemiology of tongue lesions within the Italian population. In this report, the histopathological and clinical appearance of 1.106 tongue lesions from northern Italians are described and discussed.

**Material and Methods:**

The case records of patients referred for the diagnosis and management of tongue lesions, from October 1993 to October 2013, were reviewed. Histological data were also obtained and blindly reexamined.

**Results:**

For instance, a biopsy performed on a lingual ulcer has a strong predicting association with a carcinoma, whereas a biopsy on a white lesion predicts for a leukoplakia or oral lichen planus. Moreover, a biopsy of erosion is representative of bullous diseases, whereas a biopsy on a verrucous-papillary lesion is significant for fibroma. Furthermore, carcinomas occur in the majority of cases on the lingual edge or pelvis, oral lichen planus is mainly seen on the edge, and fibromas mostly on the lingual tip.

**Conclusions:**

The high frequency of tongue involvement of such different diseases emphasizes the importance of histological characterization and that some diseases occur more frequently than others, with a peculiar clinical aspect and a more common area. In fact our survey can help the clinician in advancing diagnostic hypothesis, on the basis of the elementary lesion and its site of involvement.

**Key words:**Tongue lesions, clinical appearance, histological description.

## Introduction

Medical literature offers no many surveys investigating the rate of lingual diseases following biopsy. Most researches analyzed more than so-called pseudo-diseases which are diagnosed by means of a clinical examination (1-3), or else, involving biopsies, concentrate on the whole oral mucosa (4).

The tongue is, in fact, commonly affected by non-neoplastic and neoplastic lesions, the latter usually being characterized by a progressive growth that can be either benign or malignant. Non-neoplastic lesions are either inflammatory or represent a reaction to diverse types of irritative stimuli and are often discovered accidentally during routine oral examination (5). Nevertheless, the baseline data on tongue lesions is necessary for oral health planning and education and is of clinical and therapeutic importance for oral/ dental health care providers. Despite the abundance of the worldwide surveys on the prevalence of tongue lesions, reviewing the literature revealed the lack of studies that explored whether the affected subjects were aware of the existence of their tongue lesions. In addition, the proportion of the subjects’ perception of symptoms and their knowledge about these commonly encountered tongue lesions has not been investigated, nor the treatment attempted for the management of these lesions by the general dental practitioners (6). An accurate comparison and evaluation of the findings obtained by previous epidemiological studies is difficult because of the limited number of reported cases; this is the reason why estimating the prevalence of tongue lesions worldwide proves to be an arduous task.

Our aim was to evaluate the correlation between the clinical variables taken into consideration (i.e. involvement site and elementary lesion) and each individual disease of tongue lesions in a wide northern Italian population.

## Material and Methods

The case records of all patients, who had been initially referred to the Oral Medicine Unit of the main hospital of the city of Turin, Italy, for the diagnosis and management of lingual lesions, from October 1993 to October 2013, were reviewed and relevant retrospective data selected and extracted. Demographic information, age and gender, smoking, alcohol consumption, clinical aspect of the lesions, and sites of oral involvement were collected. To complete the final list, the following inclusion criteria were adopted: 1) all age groups and both sexes; 2) reports with complete and satisfactory case histories; 3) more than one sample for a given patient, as long as biopsies at different times.

Data regarding the histological type of lesion were obtained from the biopsy register for each case. Haematoxylin and eosin sections of each specimen were evaluated by light microscopy and blindly reexamined by an expert oral pathologist.

All tongue disease, found in our sample, have been identified on the corresponding elementary lesion described in clinical observation, being: a) white lesions, b) white-red lesions, c) red lesions, d) verrucous-papillary lesions, e) exophytic lesions (lumps), f) submucosal nodes (bumps), h) pigmented lesions, i) red-blue lesions, l) ulcers and m) erosions. We usually described an ulcer as a loss of epithelium due to any cause, while we used the term erosion to report a superficial defect producing some loss of epithelium; however, for practical purpose, those terms can be used interchangeably.

Subsequently, according to histological diagnosis and clinical appearance, the diseases were classified into 4 groups by two expert oral physician (M.C., P.G.A.): a] neoplastic lesions (either malignant or benign); b] non-neoplastic lesions (including traumatic, infectious, pseudopathologies and not otherwise defined lesions); c] oral pre malignant lesions; d] lesions caused by autoimmune diseases.

The statistical analysis of results was performed by determination of the Odds Ratio (95% Confidence Interval), in order to assess the degree of association between clinical parameters considered (site, elementary lesions) and each pathology. The statistical evaluation was made by Mantel-Haenszel test and were considered significant for *p* values <0.05. All analyses were performed using SPSS- software (SPSS for windows, version 11, SPSS inc, Chicago, IL, USA).

Different treatment options were discussed with the patients, and they all submitted written informed consent before biopsy, which was carried out in accordance with the declaration of Helsinki. The study was also approved by the Ethical Committee of the CIR - Dental School, University of Turin.

## Results

The study group involved 627 females and 479 males (F: M = 1.3: 1). The mean age at presentation was 55 years for men, and 59 years for women.

We investigated the frequency of 1.106 samples in both sexes, according to the lingual area sampling (tip, lateral boarder, pelvis and dorsum) and according to the clinical presentation of the elementary lesions ( Table 1).

**Table 1:**
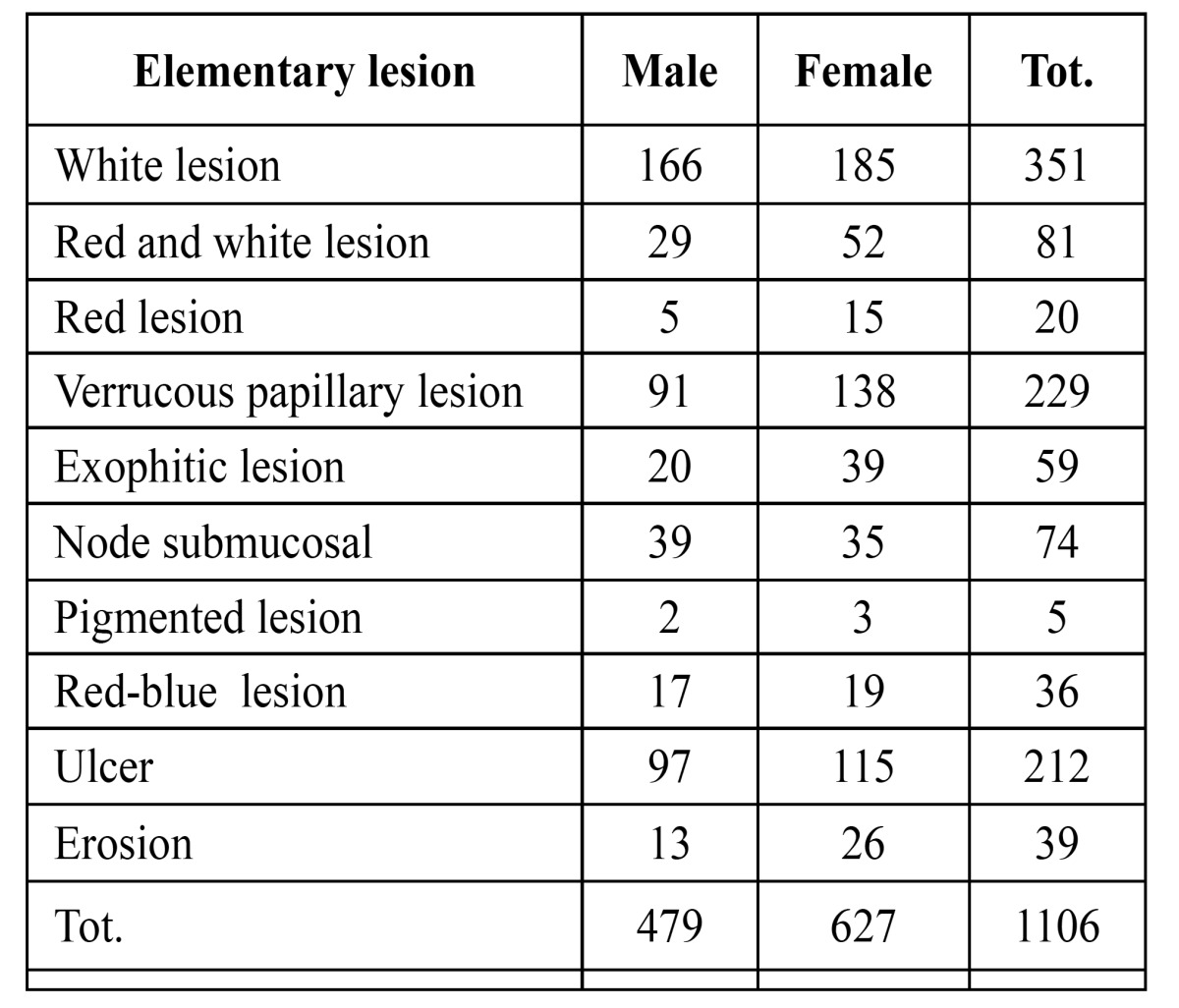
Frequency and distribution of elementary lesions.

- a. Neoplastic lesions ( Table 2).

**Table 2:**
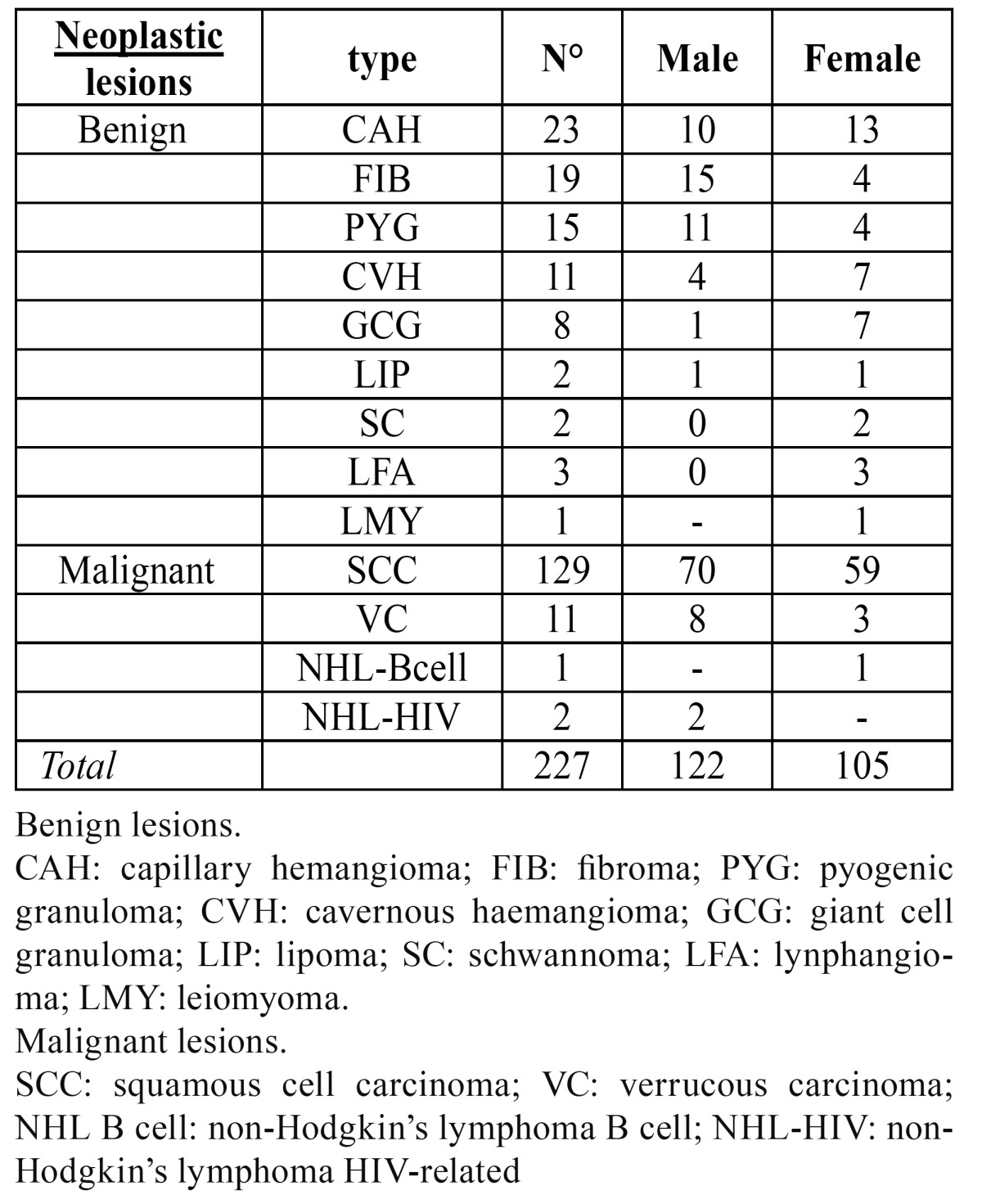
Frequency and sex distribution of neoplastic lesions.

Malignant neoplastic lesions accounted for almost 12.92% of all biopsied tongue lesions. One hundred and twenty-nine out of 143 malignant tumours were diagnosed as oral squamous cell carcinoma (89%). Among other malignant neoplasms, we found verrucous carcinoma (8%) and non-Hodgkin lymphomas (3%). When considering sampling sites, the majority of surgical samples were obtained from the lateral boarder (68%), followed by pelvis (21%), dorsum (8%) and tip (3%).

Malignant lesions manifested as ulcers in the vast majority of our sample (69% of cases), followed by red and white lesions (8%), withe lesions (8%), verrucous-papillary lesion (6%), erosions (4%) and others lesions (nodes, red-blue lesions, exophytic lesions) had a frequency about 2% each.

The highest number of malignant lesions was detected in patients aged 70 to 79.

Benign neoplastic lesions were classified as capillary hemangioma (8%), fibroma (7%), pyogenic granuloma (6%), cavernous haemangioma (5%), granulous cell tumour (3%), schwannoma (2%), lymphangioma (1%), leiomyoma (1%), lipoma (1%). The most commonly involved localisation resulted to be the lateral border with 36% of sample and the dorsum with 35%. A higher incidence of benignant neoplasm occurred in the age group 60-69.

- b. Non-neoplastic lesions ( Table 3).

**Table 3:**
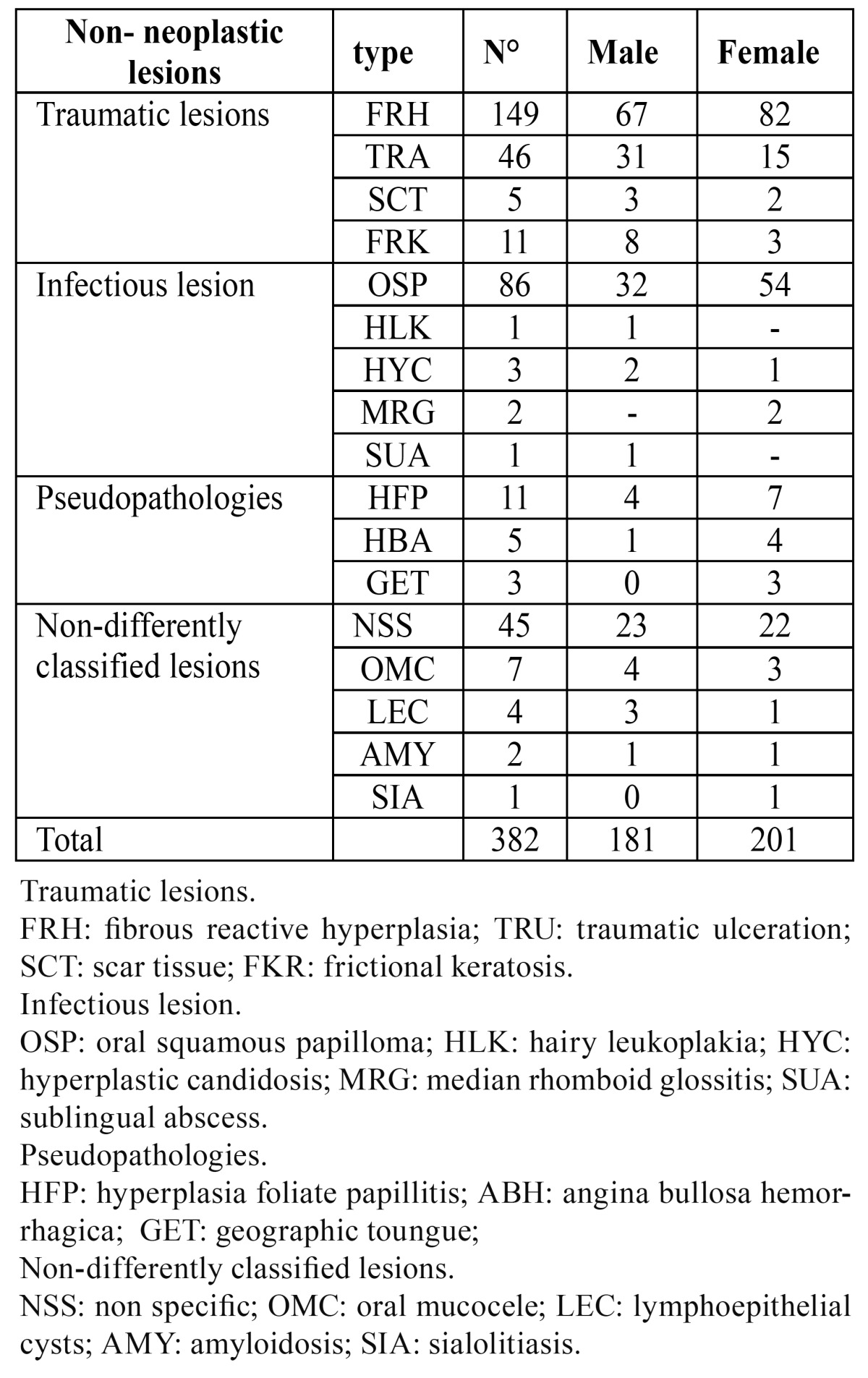
Frequency and sex distribution of non-neoplastic lesions.

Pseudo-pathologies, traumatic and infectious lesions and other pathologies belong to this group. The traumatic lesions were classified as follows: fibrous reactive hyperplasia (71%) traumatic ulcerations (22%) scar tissue (5 %) and frictional keratosis (2%). The border of the tongue is the most commonly involved site and the white lesion represented the majority of the total cases.

Infective lesions were detailed as: squamous papilloma (91%), hairy leukoplakia (4%), hyperplastic candidiasis (3%), median rhomboid glossitis (1%), and sublingual abscess (1%). The lateral border was the most commonly involved site and the verrucous-papillary-lesion represented the majority of elementary lesion.

Pseudo-pathologies occurred as follows: hyperplasia of foliate papilla (60%), angina bullosa hemorrhagica (25%), and geographic tongue (15%). The dorsum was the most commonly involved site and the exophytic lesion represented the majority of elementary lesions. Amongst the other encountered pathologies we diagnosed undifferentiated, nonspecific sample (78%), oral mucocele (12%), lymphoepithelial cysts (5%), amyloidosis (3%), and sialolithiasis (2%). The lateral boarder was the most commonly involved and submucosal node represented the majority of elementary lesion biopsied.

The non-neoplastic lesions reached a peak of incidence around the sixth decade of age, even though these lesions can be detected at any age.

- c. Oral potentially malignant lesions

OPMLs represented almost 23% of all lesions (299 overall). The following encountered lesions belong to this group: leukoplakia without dysplasia 65%, leukoplakia with mild dysplasia 18%, leukoplakia with moderate dysplasia 11%, leukoplakia with severe dysplasia 4%, and verrucous leukoplakia 2%. The elective localisation of such lesions is the lateral border (67.6%). The majority of lesions to undergo a biopsy and to obtain the subsequent diagnosis of leukoplakia were white lesions (65%), and the lesions that obtained the diagnosis of dysplasia were all exophytic in appearance. When considering age distribution, premalignant lesions showed an incidence peak around the sixth decade.

- d. Oral inflammatory diseases

Oral lichen planus showed to be the most frequent pathology, representing 57% of all disimmune diagnoses, followed by oral lichenoid lesions (19%), aphthous ulcers (9%), lupus erythematosus (6%), mucous membrane pemphigoid, erythema multiforme, Bechet’s disease (2%), pemphigus vulgaris and graft versus host disease (1%). The lateral border was the most commonly involved tissues (63%), followed by the dorsum (17.2%), pelvis (16.7%) and tip (3.5%). White lesions were the most represented in this group (59%), and the incidence resulted to be higher in the seventh decade.

 Table 4 shows the statistically significant relationships occurred from our data analysis. Such investigation aimed at focusing on the association between clinical aspect of the lesion, histopathological diagnosis and site of biopsy. Our sample demonstrated how lingual ulcers have a strong positive association with cancer, trauma or dysplasia, whereas white lesions are associated with leukoplakia or oral lichen planus. Finally, our data showed that erosions have a strong positive correlation with bullous diseases, especially oral lichen planus.

**Table 4:**
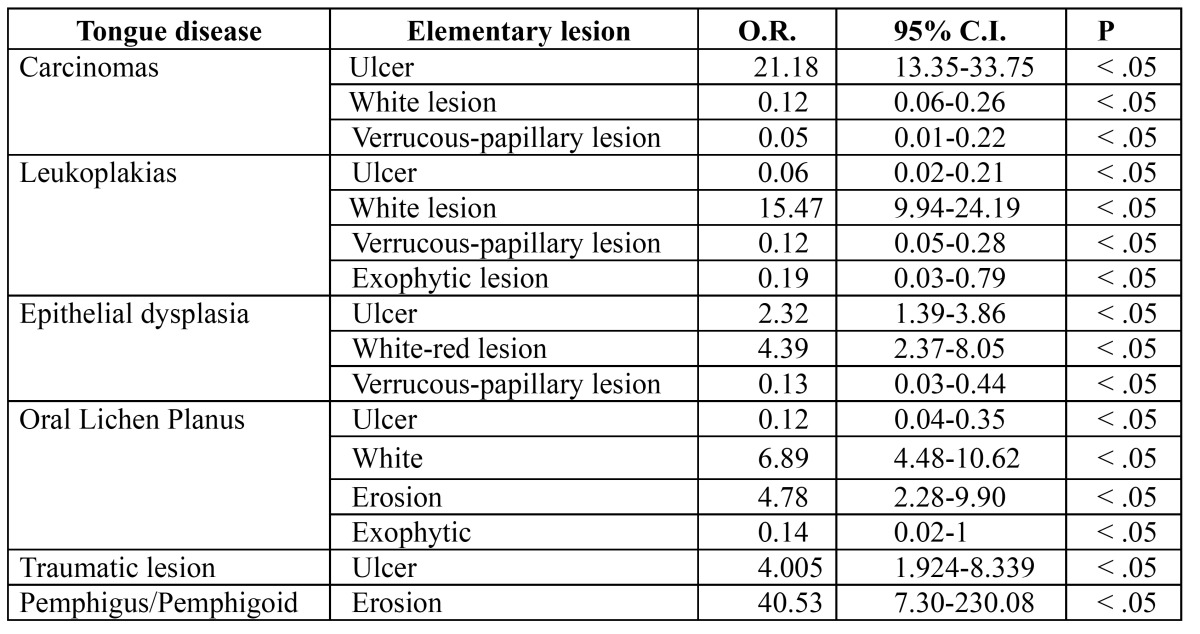
Clinical pathological correlation.

## Discussion

To the best of our knowledge, this study represents the first report on the frequency and distribution of biopsied lingual lesions within the northern Italian population. Few studies investigate the frequency of oral diseases exclusively on tongue.

A recent study (7) investigated the frequency of tongue’s diseases followed by biopsied in an Iranian population, but to date, there aren’t surveys that have correlated clinical appearance to histopathological features. Some studies mainly focus on the diagnosis of pseudo-pathologies by means of clinical inspection (1-3). A number of works examines data regarding the whole mucosa of the mouth thus including some data regarding the tongue (4). Similarly, studies that analysed merging data from clinical exam and biopsy when needed, included data from the whole oral mucosa (8-10).

Our sample showed a prevalence of lingual lesions in females; this is in agreement with results obtained by other studies (7,11-14). The study by Avacu (3) considered uniquely the tongue and highlighted a statistically significant correlation with male gender, and increasing age while Alaeddini (7) report the mean age (47 years) did not differ between male and female subjects with tongue’s diseases. Our sample showed age frequency peaks: this occurred in the sixth decade for males (age 50-59) and in the seventh decade for females (age 60-69).

The majority of tongue pathologies belong to one of the following: pre cancerous lesions (28%), disimmune pathologies (19%), benign (16%), and malignant neoplastic lesions (15%). These results are similar to those obtained by Alaeddini (7), although he does not have the distinction in groups of diseases: in fact, the most common lesions highlighted in the Iranian population were: lichen planus, irritation fibroma, squamous cell carcinoma and pemphigus vulgaris.

**Figure 1:**
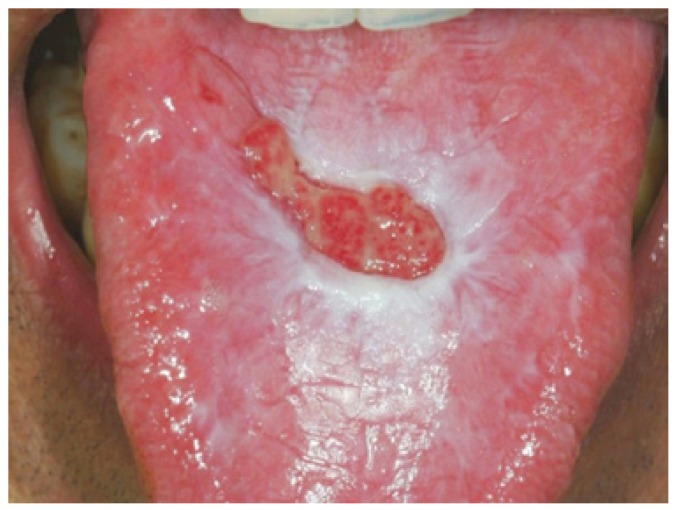
Oral lichen planus in dorsum of toungue.

**Figure 2:**
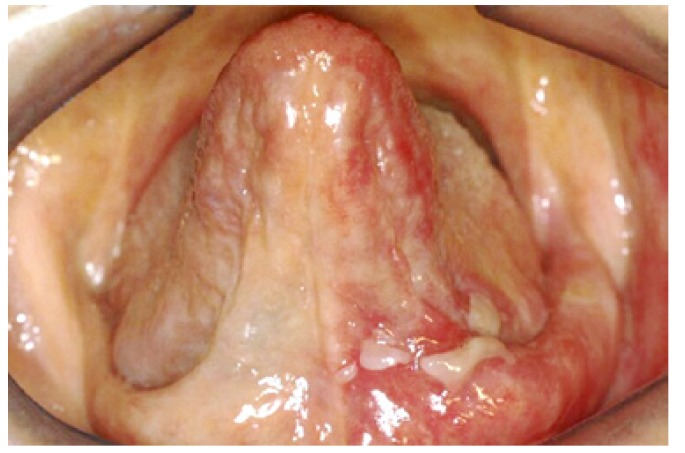
Vulgar Pemphigus in pelvis of toungue.

In agreement with this, we note that disimmune pathology most frequently encountered in our sample is oral lichen planus (Fig. 1). Such disease has the largest incidence amongst all disimmune lesions affecting the entire oral cavity and shows a peak in incidence between the sixth and seventh decade. One has to bear in mind that the majority of disimmune pathologies contemporarily involve multiple mucosal sites thus meaning that the clinician in charge of carrying out the biopsy is usually influenced by diverse factors when choosing a suitable site. This is the reason why it proves difficult to establish a correct frequency: patients who showed erosive or bullous lingual lesions (Fig. 2) may have been biopsied in another oral site because of the simultaneous involvement of different parts of the mucosa. Fronie and co-workers (15). have analysed benign and malignant neoplasias but considered a limited number of samples of mesenchymal tumours.

Our analysis showed that 70% of biopsies was performed on the tongue margin and on the ventral surface. It is implied that, within this study, most lesions occur in sub-sites where mucosa is thinner. This appears similar to what has been reported by Al-Mobeeriek and co-workers (11), who described their specimens as belonging mostly to the tongue margin.

Surely the most interesting and original data from our analysis is the clincopathological correlation of tongue lesions. The association between the elementary lesion that undergoes a biopsy, the biopsy site and the subsequent histopathological diagnosis highlights the fact that certain tongue diseases occur more frequently than others, and that they show a specific pattern in a specific preferred site.

**Figure 3:**
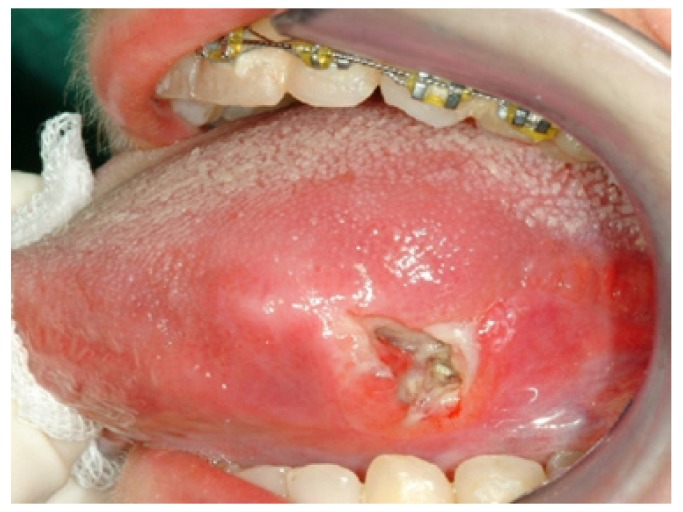
Squamous cell carcinoma in border toungue.

**Figure 4:**
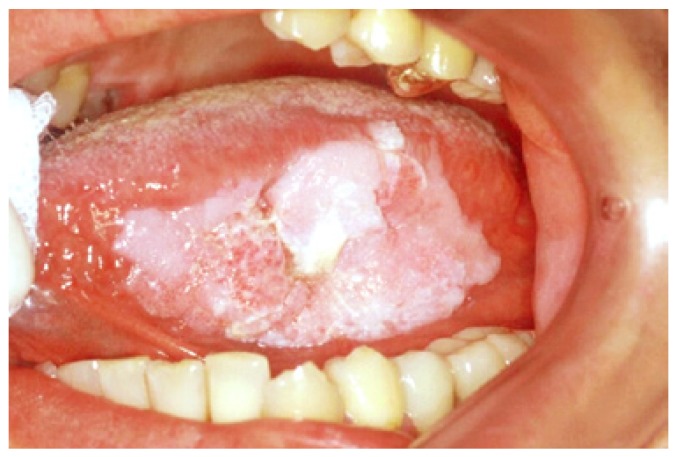
Leukoplakia with mild dysplasia in border toungue.

Elementary lesions that were more often detected and examined are colour variation areas (41.2%), lesions by excess (31.5%) and lesions by defect (27.3%). In our case series an ulcer on the tongue margin was representative of squamous cell carcinoma in 80% of cases (Fig. 3). This is in agreement with a recent study (16) which shows a high relationship between ulcer on the tongue and squamous cell carcinoma, which is often a negative prognostic factor: non-healing ulcers and oral pain were the most common reasons for the first clinical examination, very often in an advanced tumour stage. The white lesions are strongly associated with leukoplakia (Fig. 4) (OR 15.45, C.I. 9.94-24.19).

In this series the occurrence of tongue pseudo-pathologies shows a very low incidence, with 3 cases only of geographic tongue. This is in contrast with what was found in the literature and is explained by the fact that pseudo-pathologies are normally not subject to biopsy with the exception of rare diagnostic doubts. A final thought has to be given to especific histological findings: these represent 3.6% of biopsies, thus demonstrating that making a proper diagnosis can at times be difficult even when clinical and histopathological data are meticulously analyzed.

In conclusion, tongue lesions constitute a considerable proportion of oral mucosal lesions, and are a health concern to both oral health care providers and the public. Due to the widespread diffusion of these conditions, general dental practitioners and dental health care providers should be fully aware about the diagnosis, aetiology and the appropriate management of these lesions and conditions using histology.

The epidemiological studies have shown variable prevalence rates in different parts of the world (1-5). Repeatedly, the difference in the preva¬lence rates has been related to ethnic or racial factors, smoking habit and gender differences between populations studied, in addition to the general health status and the diagnostic criteria used: in fact more prospective studies are needed to better describe the real incidence of tongue diseases.
